# One case of percutaneous lung biopsy assisted by artificial pneumothorax technique and literature review

**DOI:** 10.1097/MD.0000000000046418

**Published:** 2025-12-26

**Authors:** Jiahao Li, Qiuyan Fan, Yao Dai, Yuheng Zhang, Yulong Zhu

**Affiliations:** a The Fourth Clinical Medical College of Xinjiang Medical University, Urumqi, China; b Chinese Medicine Hospital of Gao County, Yibin, China; c Xinjiang Uygur Autonomous Region Traditional Chinese Medicine Hospital, Urumqi, China.

**Keywords:** artificial pneumothorax, case report, lung cancer, percutaneous lung biopsy

## Abstract

**Rationale:**

Early lung cancer diagnosis is crucial for prognosis, but elderly patients with comorbidities poorly tolerate invasive procedures. Conventional percutaneous biopsy for major vessel-adjacent high-risk nodules has a mere 60% to 70% success rate and high complications, while artificial pneumothorax boosts it to 85% to 90%. This case verifies the technique’s safety in this population.

**Patient concerns:**

A 72-year-old female had a 7-mm left lung ground-glass nodule (2021, no intervention). Follow-up computed tomography (CT) (November 2024) showed the nodule enlarged to 10 × 10 mm with a new 18 × 17 mm irregular consolidation. Empirical anti-infection failed; the lesion was adjacent to the aortic arch and heart, so artificial pneumothorax-assisted CT-guided biopsy was adopted.

**Diagnoses:**

Video-assisted thoracoscopic surgery confirmed stage IA1 (tumor, node, metastasis stage T1aN0M0) lung adenocarcinoma (lepidic-predominant + invasive mucinous subtypes), with negative margins and no vascular, nerve, or pleural invasion.

**Interventions:**

CT-guided artificial pneumothorax-assisted percutaneous lung biopsy, followed by video-assisted thoracoscopic surgery left upper lobectomy plus lymph node dissection.

**Outcomes:**

Vital signs were stable; drainage tube removed on day 4 post-biopsy. One-month follow-up: no hemoptysis, chest pain, or pneumothorax; CT showed no intrapulmonary exudation.

**Lessons:**

The technique is safe for lesions adjacent to the heart and major vessels. However, large-sample studies and artificial intelligence–based nodule analysis are needed to further verify its efficacy.

## 1. Introduction

Lung cancer early diagnosis is key to improving prognosis. With global aging, elderly patients with comorbidities (e.g., hypertension) are common, yet conventional percutaneous lung biopsy for high-risk nodules (near major vessels) has low success (60%–70%) and high complications. Artificial pneumothorax, via controlled lung collapse, boosts biopsy success to 85% to 90% and reduces risks. This study explores its value via a 72-year-old patient case to guide clinical practice.

## 2. Clinical data

### 2.1. Patient characteristics

In 2021, a 72-year-old female’s chest computed tomography showed a 7 mm ground-glass nodule in the left upper lobe anterior segment, with no subsequent intervention. In November 2024, follow-up chest computed tomography revealed the left upper lobe ground-glass nodule had enlarged, plus an 18 × 17 mm consolidation in the left lingular segment. She had hypertension for over 30 years, no smoking/alcohol history, and no family tumor history.

Physical exam: temperature 36.6 °C, pulse 87 bpm, respiratory rate 19 bpm, blood pressure 128/91 mm Hg. Respiratory movements: increased, regular rhythm; intercostal spaces normal. Bilateral vocal fremitus: symmetrical; no pleural friction rub. Lung percussion: bilateral resonance; breath sounds: increased, no rales/pleural friction rub/prolonged expiration. Vocal resonance: symmetrical.

### 2.2. Related auxiliary examinations

**Figure 1.**
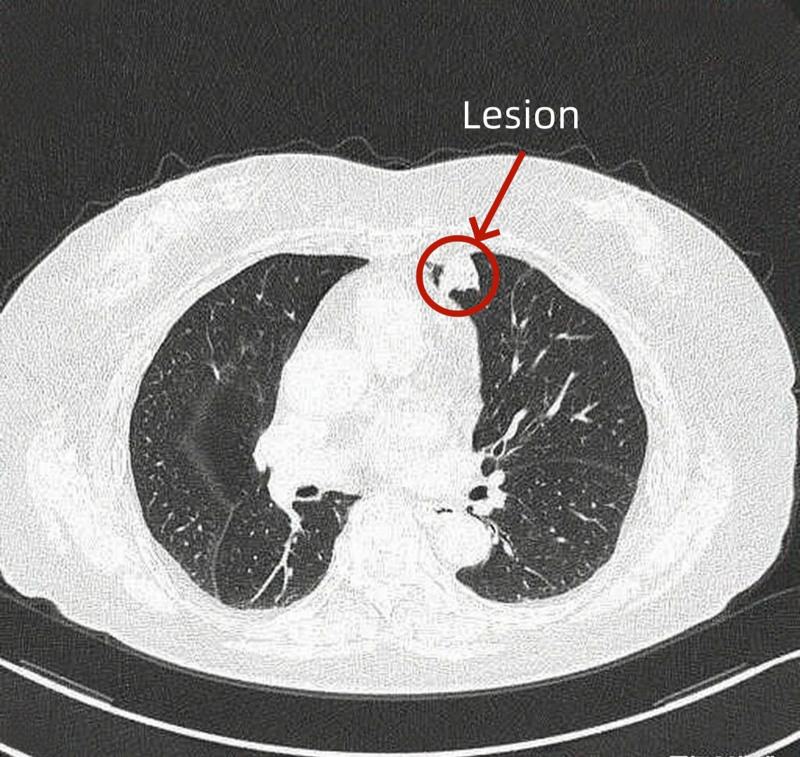
High-resolution computed tomography (HRCT) of the lungs in November 2024.

On admission (25 November, 2024), chest computed tomography showed a 10 × 10 mm ground-glass nodule and an 18 × 17 mm high-risk irregular nodule in the left upper lobe, multiple small solid nodules (largest 6 × 5 mm in the right horizontal fissure), aortic sclerosis, right breast calcification, and hepatic patchy low-density lesions with calcification (suggesting further evaluation) (Fig. 1). Blood routine revealed neutrophilia (65.4%, 4.52 × 10^9^/L) with normal other cell counts; virus serology showed IgG(+)/IgM(‐) for herpes simplex virus, cytomegalovirus, rubella, and Epstein–Barr virus. Blood gas, tumor markers (carcinoembryonic antigen, cytokeratin 19 fragment antigen 21-1), rheumatological/immunological panels, liver/kidney function, and infection screens (tuberculosis antibody, purified protein derivative, sputum examination for acid-fast bacilli, galactomannan test) were all normal. Based on the patient’s current relevant blood test results and comparison of imaging findings, the nature of the patient’s space-occupying lesion is highly likely to be a malignant tumor; however, infection cannot be ruled out. Therefore, empirical treatment with antibacterial and antiviral drugs was administered. No significant reduction in the space-occupying lesion was observed, so further performance of a puncture biopsy is required to confirm the diagnosis. Considering the patient’s advanced age and the fact that the space-occupying lesion is located close to the heart, our team has opted for the assisted artificial pneumothorax technique.

### 2.3. Artificial pneumothorax-assisted percutaneous lung biopsy and postoperative pathological results

1. Preoperative preparation

*Preoperative assessment:* Preoperative examinations indicated that the patient had a bacterial–viral coinfection.

*Preconditioning regimen:* Empirical anti-infective therapy was administered, specifically including intravenous infusion of levofloxacin and oral administration of oseltamivir, with a total course of 4 days.

*Proposed surgical plan:* It was planned to perform “computed tomography-guided percutaneous lung biopsy assisted by artificial pneumothorax” under local anesthesia.

*Preoperative patient preparation:* The patient had normal coagulation function. Anticoagulant drugs were discontinued 1 week prior to surgery, and bridging therapy with low-molecular-weight heparin was initiated. Low-molecular-weight heparin was discontinued 24 hours before surgery. The patient was informed that the supine position would be required during the operation, and was instructed to practice maintaining this position for 30 to 60 minutes to avoid movement due to positional discomfort during the operation (which might affect puncture accuracy). Meanwhile, the patient was also guided to practice calm breathing and breath-holding.

1. Intraoperative process

*Preparation for basic operations:* The puncture site was determined as the anterior axillary line (avoiding the inferior margin of the rib). Skin disinfection was performed over a 15 cm range, and local anesthesia was administered using 2% lidocaine up to the pleural layer.

*Establishment of artificial pneumothorax:* A total of 180 mL of filtered air was injected through a coaxial pneumothorax needle under negative pressure. Vital signs were continuously monitored during the operation: oxygen saturation ranged from 92% to 97%, heart rate ranged from 83 to 88 beats per minute, and blood pressure ranged from 137–147/86–92 mm Hg.

**Figure 2.**
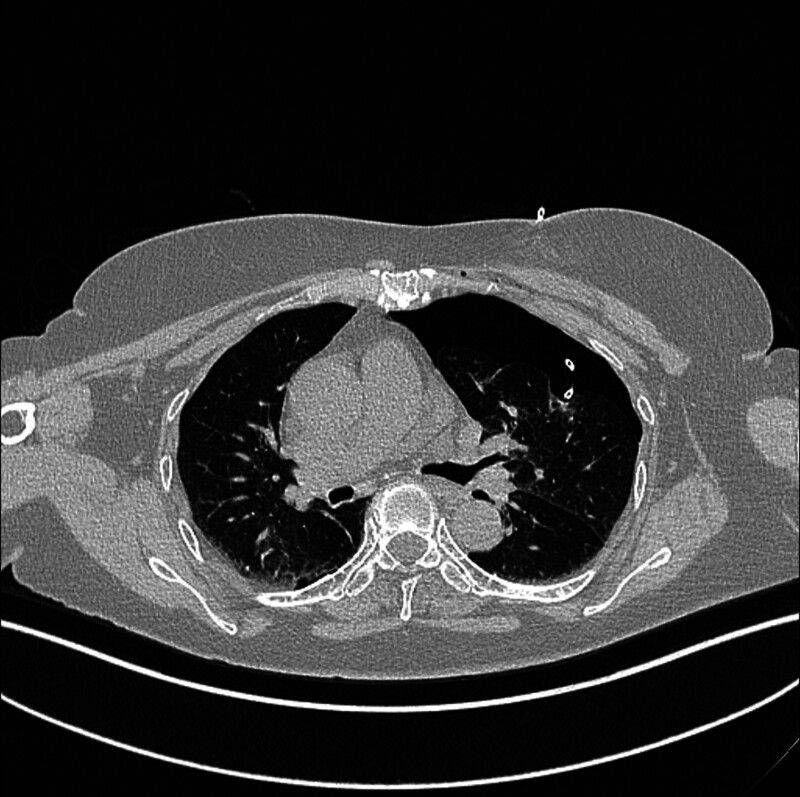
Artificial pneumothorax procedure.

**Figure 3.**
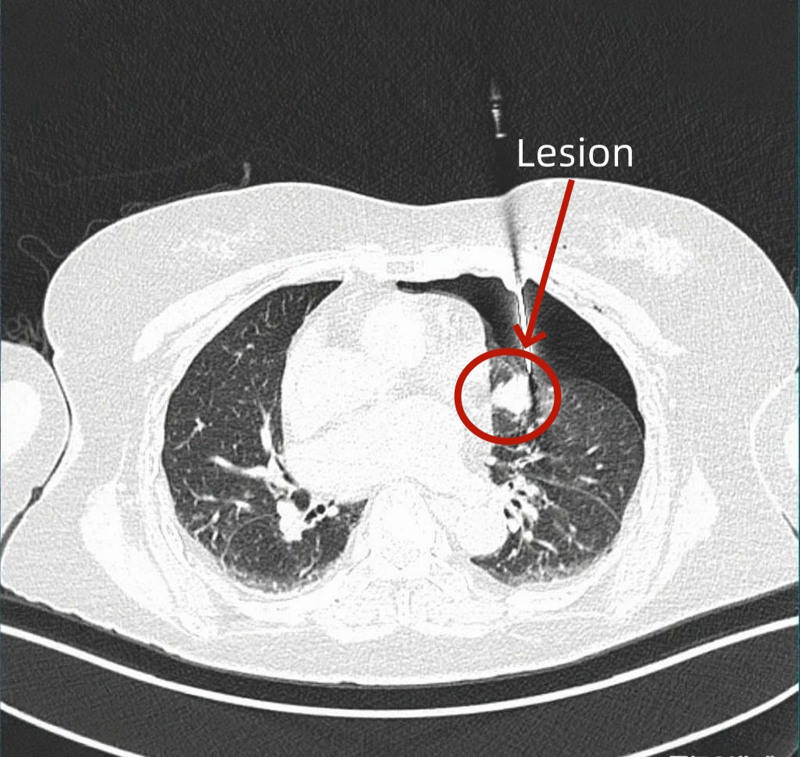
Artificial pneumothorax-assisted percutaneous lung biopsy.

*Imaging confirmation and core operations:* Chest computed tomography examination confirmed 30% compression of lung tissue and successful nodule displacement (Fig. 2), followed by placement of a drainage tube. Lesion specimens were obtained using a coaxial biopsy needle (Fig. 3).

**Figure 4.**
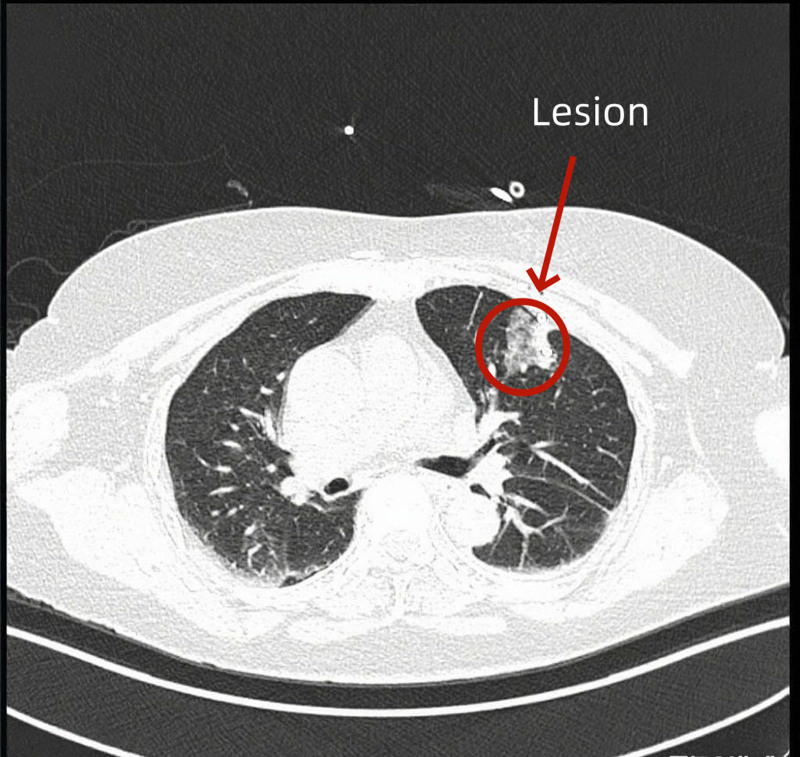
After suctioning gas from the artificial pneumothorax.

*Intraoperative immediate management and short-term postoperative management:* Gas in the artificial pneumothorax was aspirated to promote lung reexpansion (Fig. 4). Daily chest X-ray examinations were performed postoperatively. After imaging showed no effusion or gas, the drainage tube was removed on the 4th postoperative day.

1. Postoperative pathology

*Initial puncture pathology result:* Microscopically, fibrinous exudate and red blood cells were observed, with no malignant tumor cells detected.

*Indication for surgical intervention:* Comparison with imaging data in 2021 indicated an increase in nodule volume. After consultation with the patient’s family, it was decided to further perform surgical treatment.

*Pathology result of surgically resected specimens:*

Surgical conditions: Under combined IV-inhalational general anesthesia via left anterior chest, video-assisted thoracoscopic surgery lobectomy was performed. Intraop, left upper lobe bronchus was dissected, with lymph node dissection for stations 4L/5/6/7/8/9/10/11. Intraop blood loss was ~100 mL. One thoracic drainage tube was indwelled postop. The patient was in intensive care unit for 1-day transition, and safely transferred to general ward after successful ventilator weaning.

Lesion nature: The small lesion was lepidic-predominant adenocarcinoma, and the large lesion was invasive mucinous adenocarcinoma.

Tumor staging: Stage IA1 (T1aN0M0).

Surgical margin: Negative surgical margin.

Invasion status: No vascular invasion, no nerve invasion, and no pleural invasion.

*Immunohistochemistry results:*

Small lesion: Thyroid transcription factor 1 positive, Napsin A positive.

Large lesion: Thyroid transcription factor 1 focally positive, cytokeratin 7 positive, cytokeratin 20 positive.

Diagnostic conclusion: Combining the above pathological and immunohistochemical results, the patient was diagnosed with lung cancer.

At the 1-month follow-up after surgery, the patient underwent a chest computed tomography reexamination, which showed no obvious intrapulmonary exudation. The patient had no adverse reactions such as hemoptysis, chest pain, or pneumothorax, and the follow-up is still ongoing.

## 3. Discussion

Precision diagnosis/treatment of pulmonary diseases is crucial. With advances in percutaneous biopsy and minimally invasive ablation, artificial pneumothorax-assisted technique aids difficult cases.^[1]^ A 72-year-old female had a left lung lingular lesion (rib-covered, adjacent to great cardiac vessels)— high-risk for traditional procedures. Poor age-related tolerance led to using this technique: lesion displaced, risks reduced, specimen obtained successfully without severe complications.

The artificial pneumothorax technique originated in the late 19th century, initially used to treat tuberculosis. Although domestic thoracic surgery guidelines do not dedicate a separate chapter to it, it is mentioned in sections on pleural effusion and pulmonary mass biopsy, serving as bridge therapy for recurrent pneumothorax or assisting in localization during pulmonary mass biopsy to improve sampling success rates.^[2,3]^ In the absence of pleural effusion, it reduces blind spots in thoracoscopic visualization from 30% to 40% to < 10% and improves the positive rate of percutaneous pleural biopsy by approximately 25%.^[4]^

Common complications of artificial pneumothorax-assisted techniques include pneumothorax recurrence (10%–15%, related to underlying pulmonary diseases and pleural adhesions), subcutaneous emphysema (pin-tract air leakage, self-resolving in mild cases), mediastinal emphysema (<5%, requiring urgent decompression),^[5,6]^ and tumor seeding along the needle tract (0.02%–0.06%, reducible via strict no-touch neoplasm principles),^[7]^ as well as microwave ablation-related complications such as local pain (40%–50%), fever (30%), infection (<5%), and neurovascular injury (<3%) that require targeted prevention and control.^[8]^ The promotion of this technique faces multi-dimensional challenges: it highly relies on operator experience, as precise localization and needle insertion require profound anatomical knowledge—novice operators are prone to inappropriate pneumothorax volume or lung tissue injury, while experienced physicians may still face challenges from complex anatomical variations^[9]^; inadequate precision of gas injection equipment compromises pneumothorax stability, and suboptimal resolution or real-time performance of imaging guidance equipment increases the risk of misjudgment^[10]^; gas injection disrupts the intrathoracic environment, where residual air or rapid carbon dioxide absorption may cause delayed lung reexpansion or respiratory acidosis, threatening elderly patients or those with compromised cardiopulmonary function^[11]^; and lung collapse may lead to ventilation-perfusion mismatch or atelectasis, with long-term or repeated use cumulatively impairing lung function and accelerating respiratory deterioration in patients with preexisting poor pulmonary reserve.^[12]^

The artificial pneumothorax-assisted technique is in the emerging stage with rare clinical use, so procedural safety demands attention. Intraoperatively, conduct continuous electrocardiogram monitoring, record vital signs (heart rate, blood pressure, and oxygen saturation) every 5 to 10 minutes (address abnormalities promptly), monitor respiratory rate, and administer oxygen to keep oxygen saturation >90%.^[13]^ Postoperatively, closely observe pneumothorax for 3 days: secure drainage tube to prevent kinking, regularly assess drainage fluid (volume, color, and properties), and replace drainage device/dressing daily.^[14]^ Watch for complications (recurrent pneumothorax (10%–15%), hemopneumothorax, subcutaneous emphysema, infection) with hierarchical management: conservative treatment for mild hemopneumothorax (surgery for massive cases); observation for mild subcutaneous emphysema (aspiration/decompression for severe); anti-infective therapy for infection (adjust meds per antibiotic susceptibility test).^[15]^

The artificial pneumothorax-assisted technique is crucial for the diagnosis and treatment of complex pulmonary diseases. When lesions are adjacent to vital structures such as the mediastinum, heart, and large blood vessels, the operational risk is significantly reduced. This case suggests that the artificial pneumothorax-assisted technique greatly improves the safety of percutaneous lung puncture performed in relatively complex intrapulmonary locations, and supplements evidence for its clinical application in this population. However, due to the single-case design of this study, as well as the lack of long-term follow-up and follow-up of clinical data, subsequent studies may conduct follow-up of relevant data (e.g., serum calcium ions, modified Glasgow Prognostic Score). Additionally, the role of artificial intelligence in the differentiation of pulmonary nodules requires further exploration.^[16–18]^

## Author contributions

**Conceptualization:** Jiahao Li, Yulong Zhu.

**Data curation:** Qiuyan Fan.

**Formal analysis:** Qiuyan Fan, Yao Dai, Yuheng Zhang.

**Methodology:** Yao Dai.

**Visualization:** Yuheng Zhang.

**Writing – review & editing:** Yulong Zhu.

**Writing – original draft:** Jiahao Li.

## References

[1] Yang CQ Du RH Cao TZ. A case of CT-guided percutaneous closed pleural biopsy after artificial pneumothorax. Proceedings of the 2019 National Academic Congress on Tuberculosis, Tuberculosis Society of Chinese Medical Association, Suzhou, Jiangsu, China, 2019.

[2] Jian L Chunhai L Liangzhong L Xi C Minggui Z. CT-guided percutaneous biopsy of lesions beside heart blood vessels or in the mediastinum after establishing artificial pneumothorax. Chin J Interventional Imag Ther. 2020;17:763–5.

[3] Shanjun P Jun Y Yaozeng X Shuqin P Fengquan L. Clinical discussion on different methods of CT-guided puncture biopsy of chest lesions. Chin J Interventional Imag Ther. 2007;05:348–50.

[4] Shijie Y Jianchuan D. Comparison of efficacy and safety of thoracoscopic surgery with or without artificial pneumothorax in the treatment of mediastinal tumors. Med Forum. 2022;26:47–9.

[5] Junjun H Yan H Xiangdong M. Thoracic ultrasound versus artificial pneumothorax in complications of medical thoracoscopy – a propensity score matching analysis. Thorac Dis. 2018;10:5269–74. 10.21037/jtd.2018.08.41 PMC6196214 30416774

[6] Yunyan W Congcong Z Xinshan L. Safety and complications of medical thoracoscopy in the management of pleural diseases. BMC Pulm Med. 2019;19:125. 31291926 10.1186/s12890-019-0888-5 PMC6617601

[7] Tian J Miao L Cheng Z. High-pressure artificial pneumothorax promotes invasion and metastasis of oesophageal cancer cells. Interact Cardiovasc Thorac Surg. 2019;29:275–82. 30927432 10.1093/icvts/ivz085

[8] Shuo W. Clinical Research of Artificial Pneumothorax and Sternum Traction Hook in Modified Subxiphoid Thoracoscopic Thymectomy. Zunyi Medical University, 2021.

[9] Haiyong W Guangmao Y Bin W Linhai F. Comparison of clinical effects of single-port thoracoscopic surgery and multi-port thoracoscopic surgery in resecting mediastinal tumors. Zhejiang J Traumatic Surg. 2019;24:13–5.

[10] Shuo L. Application of artificial pneumothorax in thoracoscopic radical resection of esophageal cancer. Chin J Thoracic Cardiovascular Surg. 2017;33:439–40.

[11] Cheng Q Zhang J Wang H Zhang R Yue Y Li L. Effect of acute hypercapnia on outcomes and predictive risk factors for complications among patients receiving bronchoscopic interventions under general anesthesia. PLoS One. 2015;10:e0130771. 26147645 10.1371/journal.pone.0130771 PMC4492548

[12] Yunqin R Xiaoying F Tianyuan Z Xin Z Hong Y Qinxiang M. Research progress on the impact of artificial CO_2_ pneumothorax on the body. J Region Anat Operat Surg. 2020;29:927–31.

[13] Qingshuang Y Jingyu L. Analysis of the advantages and disadvantages of establishing artificial pneumothorax before medical thoracoscopy. China Med Eng. 2020;28:32–7.

[14] Jinbao X Quan D Min L Yuanrong T Jianfeng C. Application of artificial pneumothorax-assisted uniportal thoracoscopic thymectomy in patients with thymic tumors. China Modern Med. 2020;28:113–6.

[15] Xujian H. Application Research of CT-Guided Artificial Pneumothorax Technique in Microwave Ablation of Subpleural Lung Tumors. Shandong University, 2021.

[16] Shu Y Li KJ Sulayman S. Predictive value of serum calcium ion level in patients with colorectal cancer: a retrospective cohort study. World J Gastrointest Surg. 2025;17:102638. 40162418 10.4240/wjgs.v17.i3.102638 PMC11948136

[17] Guo W Chen Y. Comments on “The impact of artificial intelligence on the adenoma detection rate: comparison between experienced, intermediate and trainee endoscopists’ adenoma detection rate” [published online ahead of print August 8, 2025]. Wien Klin Wochenschr. doi: 10.1007/s00508-025-02596-6. 10.1007/s00508-025-02596-6 40779099

[18] Chen Y Zhang B Wang X. Prognostic value of preoperative modified Glasgow prognostic score in predicting overall survival in breast cancer patients: a retrospective cohort study. Oncol Lett. 2025;29:180. 39990808 10.3892/ol.2025.14926 PMC11843409

